# Analysis of the Sports Psychological Profile, Competitive Anxiety, Self-Confidence and Flow State in Young Football Players

**DOI:** 10.3390/sports12010020

**Published:** 2024-01-08

**Authors:** José A. Domínguez-González, Rafael E. Reigal, Verónica Morales-Sánchez, Antonio Hernández-Mendo

**Affiliations:** Department of Social Psychology, Social Anthropology, Social Work and Social Services, University of Málaga, 29071 Málaga, Spain; josedominguez@uma.es (J.A.D.-G.); vomorales@uma.es (V.M.-S.); mendo@uma.es (A.H.-M.)

**Keywords:** sport psychology, performance, youth sport, psychological skills

## Abstract

The objective of this study was to analyse the correlations among the sports psychological profile, competitive anxiety, self-confidence and the flow state of young football players. Additionally, potential distinctions based on age category, competitive level and field position were explored. This study included 328 participants divided into two groups: Group 1, 14–15 year olds, and Group 2, 16–18 year olds (M = 15.85; SD = 1.44). Data were collected by using the Psychological Inventory of Sports Execution (IPED), the Revised Competitive State Anxiety Inventory-2 (CSAI-2R) and the Flow State Scale (FSS). The findings revealed positive associations between the sports psychological profile and self-confidence, as well as with the flow state. Conversely, competitive anxiety demonstrated negative relationships with the sports psychological profile, self-confidence and the state of flow. Moreover, superior scores were observed in the analysed variables for the older age category and higher competitive level, with no notable disparities based on field positions. These results underscore the interplay of psychological factors in the performance of football players and underline distinctions among players according to their category (age and level). This emphasises the importance of scrutinising these variables in athletes to comprehend their profiles and enable targeted interventions aimed at enhancing their psychological resources for competitive scenarios.

## 1. Introduction

In recent years, there has been a growing recognition of the significance of developing psychological skills in athletes, as they constitute a fundamental element in achieving athletic success and advancement [1]. These skills empower athletes to adapt more effectively to competitive environments, even in high-stress and anxiety-inducing situations [2,3]. Notably, several extensively studied variables include group cohesion, self-control, anxiety, stress, emotional state, motivation, activation level, attention and self-confidence [4,5].

With the objective of encompassing a comprehensive array of influential factors in athletes’ performance, the Psychological Inventory of Sports Execution (IPED) was conceived [6]. This instrument evaluates various facets, including self-confidence, negative coping control, attentional control, visuoimaginative control, motivational level, positive coping control and attitudinal control [6]. The utility of this tool has been demonstrated in studies such as that of Garit et al. [7], wherein they correlated IPED factors with competitive anxiety and the injury history of high-performance athletes. In a similar vein, Reigal et al. [8] correlated the sports psychological profile (IPED) with competitive anxiety, mood and self-efficacy in beach handball players. The study concluded that the sports psychological profile has predictive capabilities for competitive anxiety, moods and self-efficacy.

The IPED has been specifically employed in the domain of football to assess the psychological variables pertinent to sports practice, both in youth and adults [9]. However, in grassroots football, there is a dearth of research on the psychological aspects influencing the development of sporting talent [10]. It is imperative to conduct such investigations on young athletes for two primary reasons. Firstly, the cultivation of psychological skills necessitates adaptation and learning over time. Secondly, the stress associated with competition can have detrimental effects on young athletes [11].

Football is a dynamic sport characterised by a myriad of interactions [12]. This dynamic can give rise to situations marked by high levels of stress and anxiety, which can adversely impact the attainment of athletes’ objectives [13,14]. Competitive anxiety, in particular, is one of the most extensively examined variables in football due to its profound influence on athlete performance [15,16]. It is defined as a negative emotional response to the demands of competition, encompassing both somatic (physiological) and cognitive (mental) components [17,18].

Another focal point of research in the realm of football is self-confidence [19,20]. This attribute exerts a significant influence on the sporting endeavours of young athletes [21,22]. Self-confidence is defined as an athlete’s belief in his or her own capacity to achieve optimal performance [23]. Consequently, athlete preparation assumes paramount importance as it directly impacts his or her level of self-confidence [21,24]. By bolstering an athlete’s psychological resources, self-confidence and self-perception can be fortified, thereby enhancing his or her capacity to adapt to training and competition [25,26].

On the other hand, in competitive contexts, the so-called flow state has been emphasized, characterised as an optimal psychological state wherein athletes can attain their highest level of performance [27]. Flow is defined as a subjective experience where athletes perceive a seamless alignment between the demands of sports practice and their ability to navigate them, creating an optimal state for sports performance [28]. Additionally, flow is associated with positive outcomes for athletes, including enhanced experience and subjective well-being, acting as a mediator between the two [28,29,30]. 

Moreover, psychological variables influencing sports performance may be influenced by factors such as the athletes’ age and their competitive level [10,31,32]. So, more experienced and higher-level athletes, having developed better psychological resources to adapt to the competitive environment, may demonstrate greater abilities to cope with the stressful situations inherent to sports [33,34]. Athletes, with their increased maturity, may perceive the demands of the game more effectively, enabling them to provide more efficient responses throughout the course of sporting activities [35].

Although multiple psychological variables have been studied in adolescent soccer players, there are few studies that have explored a broad psychological profile in this population. Furthermore, until now, no research has been found that evaluates these variables by age category, competitive level and position on the field. Given the critical importance of these variables and their impact on the well-being, performance and sports experience of young athletes, the objective of this study was to analyse the relationships among the sports psychological profile, competitive anxiety, self-confidence and the flow state in young football players. Additionally, we aimed to explore potential distinctions based on age category, competitive level and position on the field.

## 2. Materials and Methods

### 2.1. Design

This research follows an associative, comparative and predictive strategy [36]. A single evaluation of a set of psychological variables was carried out, in addition to various sociodemographic and sports characteristics of the sample.

### 2.2. Participants

The sample consisted of 328 participants divided into two groups: Group 1, 14–15 year olds, and Group 2, 16–18 year olds (M = 15.85; SD = 1.44), belonging to different sports entities in the province of Málaga (Spain). Group 1 consisted of 172 players with a mean age of 14.72 years (SD = 0.66). Group 2 consisted of 156 players with a mean age of 17.11 years (SD = 0.92). According to the competitive level (the league in which they compete), Group A (*n* = 138) was composed of the Division of Honor, National League and First Andalusian League, with an average age of 16.27 years (SD = 1.42). Group B (*n* = 190) consisted of the Second Andalusian League, Third Andalusian League and Fourth Andalusian League, with an average age of 15.55 years (SD = 1.38). Regarding the position on the field, there were 31 goalkeepers (M = 15.77; SD = 1.61), 111 defenders (M = 15.93; SD = 1.46), 89 midfielders (M = 15.84; SD = 1.47) and 97 forwards (M = 15.80; SD = 1.34). The inclusion criteria were being registered with any of the participating sports entities in the study and belonging to any age group mentioned above. The exclusion criteria were having any psychological disorders or using any medication that could interfere with the study.

### 2.3. Instruments

Psychological Inventory of Sport Performance (IPED) [6,37]. This questionnaire is the adaptation in Spanish of the Psychological Performance Inventory (PPI) [38,39] and is used to assess the athlete’s psychological profile. It consists of 42 items and seven factors: self-confidence, negative coping control, attentional control, visuoimaginative control, motivational level, positive coping control and attitudinal control. A Likert scale ranging from 1 “Almost never” to 5 “Almost always” is used to respond to this questionnaire. The internal consistency for this study (Cronbach’s Alpha) was as follows: self-confidence (α = 0.71), negative coping control (α = 0.69), attentional control (α = 0.73), visuoimaginative control (α = 0.70), motivational level (α = 0.74), positive coping control (α = 0.78) and attitudinal control (α = 0.77).

Revised Competitive State Anxiety Inventory-2 (CSAI-2R) [40]. This questionnaire is a revision of the Competitive State Anxiety Inventory-2 (CSAI-2) by Martens et al. [41] and is used to assess competitive anxiety. It consists of 17 items and three factors: cognitive anxiety, somatic anxiety and self-confidence. A Likert scale ranging from 1 “Not at all” to 4 “Very much” is used to respond to this questionnaire. The internal consistency for this study (Cronbach’s Alpha) was as follows: cognitive anxiety (α = 0.72), somatic anxiety (α = 0.78) and self-confidence (α = 0.82).

The Flow State Scale (FSS) [42]. This questionnaire assesses the optimal psychological state (flow) for sports performance. It consists of 36 items and nine factors: balance between skill level and challenge, action-awareness merging, clear goals, direct and immediate feedback, concentration on the task at hand, sense of control, loss of self-consciousness or inhibition, distortion of the sense of time and autotelic experience. A Likert-type scale ranging from 1 “Strongly Disagree” to 5 “Strongly Agree” is used to respond to this questionnaire. The internal consistency for this study (Cronbach’s Alpha) was as follows: balance between skill level and challenge (α = 0.76), action-awareness merging (α = 0.73), clear goals (α = 0.79), direct and immediate feedback (α = 0.82), concentration on the task at hand (α = 0.81), sense of control (α = 0.80), loss of self-consciousness or inhibition (α = 0.76), distortion of the sense of time (α = 0.73) and autotelic experience (α = 0.81).

### 2.4. Procedure

The sample was selected from various sports entities belonging to the province of Málaga (Spain). A nonprobabilistic and convenience sampling method was used. Firstly, permission was sought from the management of the clubs. Upon approval, the objectives and characteristics of the research were explained to each club coordinator and the team coaches. Subsequently, informed consent was obtained from the players and their parents/legal guardians, and data collection was carried out. 

Initially, a meeting was held with the players to explain the purpose of the investigation. Information was given about the types of questionnaires that had to be carried out and how they had to be completed. After that, they were sent a link to a Google Forms form containing the questionnaires they had to fill out. It took approximately 45 min to complete the set of questionnaires. Players were given contact information in case they had any questions during the process of completing the questionnaires. The research was carried out in the months of April and May of the 2022/2023 season.

Throughout the process, the ethical principles of the Helsinki Declaration [43] were respected. Furthermore, this research had the approval of the Ethics Committee of the University of Málaga (N. 9; CEUMA Registry N. 24-2023-H).

### 2.5. Statistical Analysis

The data underwent descriptive and inferential analyses. The Kolmogorov–Smirnov test was used to verify the normality of the data. Subsequently, a correlation analysis was conducted by using the Spearman’s Rho test. Then, a cluster analysis (k-means) was performed based on the FSS questionnaire variables to generate two groups and analyse the differences between them in terms of the different study variables. The Mann–Whitney U test was used to determine the differences between these clusters. Furthermore, a binary logistic regression analysis was conducted to predict membership in each of the generated groups through the cluster analysis by using the dimensions of the IPED questionnaire and the CSAI-2R as predictor variables. In the logistic regression analysis, conducted by using the Forward Conditional method, the Hosmer–Lemeshow and Omnibus tests were conducted to assess the model’s goodness of fit. SPSS software v.24 was used for data processing.

## 3. Results

### 3.1. Analysis of the Relationships among Sports Psychological Profile (IPED), Competitive Anxiety (CSAI-2R), Self-Confidence (IPED/CSAI-2R) and the Flow State (FSS) in Young Football Players

Table 1 shows descriptive statistics for the analysed variables. Additionally, the skewness and kurtosis values ranged from −0.98 to 0.65 and from −0.86 to 0.45, respectively. However, the Kolmogorov–Smirnov test indicated issues with normality. Also, Table 1 shows the correlation analyses conducted by using the Spearman test. As observed, the psychological profile (IPED) positively correlates with the self-confidence of the CSAI-2R and with the variables of the FSS and negatively with cognitive and somatic anxiety. Likewise, the dimensions of the FSS correlate negatively with cognitive and somatic anxiety, as well as positively with the self-confidence of the CSAI-2R. Only the variable time distortion in the FSS differs from the pattern of relationships of the set of variables, showing a positive relationship with cognitive anxiety and virtually no statistically significant relationships with the different factors of the IPED.

### 3.2. Cluster Analysis

**Figure 1:**
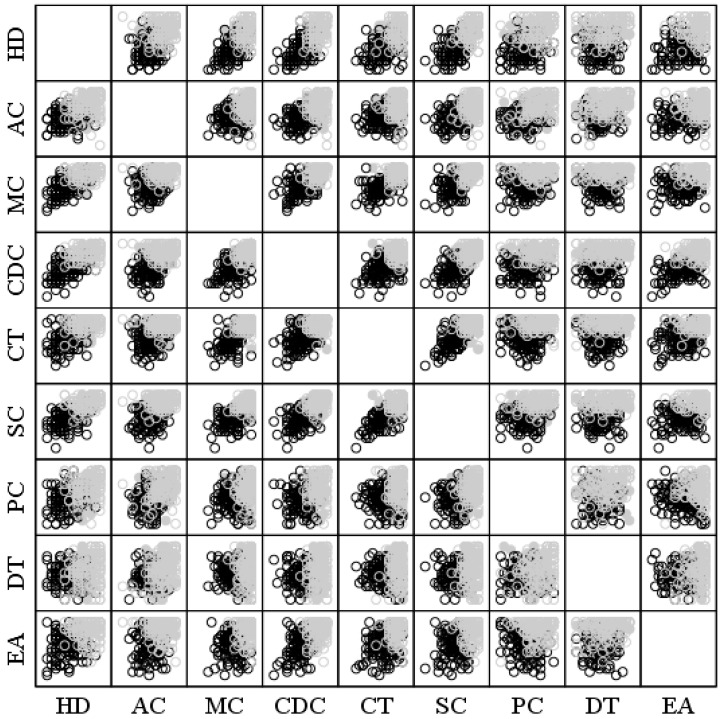
Clusters formed from the nine dimensions of the FSS. Groups: grey colour = higher level of FSS; black colour = lower level of FSS. HD = ability level and challenge; AC = action and challenge; MC = clear goals; CDC = direct and clear feedback; CT = concentration on the task; SC = sense of control; PC = loss of self-consciousness; DT = distortion of the sense of time; and EA = autotelic experience.

Two clusters were generated (k-means) from the nine dimensions of the FSS. Group 1 (*n* = 140) was characterized by lower values in the dimensions of the FSS, and Group 2 (*n* = 188) had higher scores in the FSS (Figure 1; Table 2). Each case in the clusters was well classified, given that the maximum distance (2.96) from each of them to the centre of their group was less than the distance between the centres of the clusters (3.09). Descriptive statistics of the generated clusters are shown in Table 2. Additionally, the skewness and kurtosis values ranged from −1.65 to 0.83 and from −0.99 to 2.14, respectively. However, the Kolmogorov–Smirnov tests were significant in all cases (except for negative coping control of the IPED for Group 1; K-S = 0.07, *p* > 0.05), indicating issues with normality in most of the data distributions. The conducted tests (Table 2) indicated statistically significant differences in favour of Group 2 in all factors.

### 3.3. Logistic Regression Analysis

Binary logistic regression analysis (Forward Conditional) was conducted by using the dimensions of the IPED and CSAI-2R as predictor variables and the two clusters formed from the dimensions of the FSS as the criterion variable. The internal coding of the model assumes cluster 1 (worse profile in the FSS questionnaire) as condition 0 and cluster 2 (better profile in the FSS questionnaire) as condition 1. Therefore, the generation of a statistically significant model would indicate its ability to predict membership in cluster 2 (condition 1 of the model) based on the established predictors.

The binary logistic regression analysis generated a model with four steps (Table 3). The model explained 45% of the variance (Nagelkerke R^2^ = 0.45), correctly classified 76% of the cases and had a significant Omnibus test value (χ^2^ = 134.16, *p* < 0.001) and a nonsignificant Hosmer–Lemeshow test value (χ^2^ = 5.47, *p* > 0.05), indicating a good fit of the model.

### 3.4. Analysis of the Sports Psychological Profile (IPED), Competitive Anxiety (CSAI-2R), Self-Confidence (IPED/CSAI-2R) and Flow State (FSS) Based on Age Category, Competitive Level and Position on the Field

Table 4 presents the mean and standard deviation of the study variables based on the age category and the competitive level. Additionally, the skewness and kurtosis values ranged from −1.19 to 0.79 and from −0.91 to 1.32, respectively. But, the Kolmogorov–Smirnov test highlighted issues of normality in the majority of the variables. Hence, the Mann–Whitney U test was used to analyse the differences between groups.

As shown in Table 4, the 16–18 year olds exhibited superior values in the IPED variables: self-confidence, visuoimaginative and attitudinal control. Conversely, they displayed lower cognitive anxiety and higher self-confidence in the CSAI-2R. Furthermore, they demonstrated better scores in the FSS for the variables: skill and challenge, action and awareness, direct comments and sense of control. 

On the other hand, the DH/LN/1st group displayed superior and statistically significant values in the IPED variables: self-confidence, negative coping, visuoimaginative, positive coping and attitudinal control. Moreover, they exhibited lower somatic anxiety and higher self-confidence in the CSAI-2R. Additionally, they demonstrated statistically significant better scores in the FSS for the variables ability and challenge, clear goal, direct and clear feedback, concentration on the task and sense of control.

Table 5 displays the mean and standard deviation of the study variables based on the playing position (goalkeeper vs. defender vs. midfielder vs. forward). The skewness values ranged from −1.69 to 0.99, and the kurtosis values ranged from −1.23 to 2.82. The Kolmogorov–Smirnov test indicated issues with normality in most variables. Therefore, the Kruskal–Wallis test was employed to analyse differences between groups, with no significant differences observed.

## 4. Discussion

The aim of this study was to investigate the relationships between the sports psychological profile, competitive anxiety, self-confidence and the flow state in young footballers. Additionally, we examined potential differences based on age category, competitive level and position on the field. The results revealed significant correlations among the sports psychological profile, competitive anxiety, self-confidence and the flow state. Moreover, notable distinctions were observed based on age category, favouring the junior players, and competitive level, favouring those at a higher level (DH/LN/1st). However, no statistically significant differences were found based on playing position.

Regarding the relationships among factors, positive associations were identified between the sports psychological profile, self-confidence and the flow state. This suggests that athletes with higher scores in psychological skills, as assessed by the IPED, tend to exhibit more favourable scores in variables conducive to adapting to the competitive environment, such as lower competitive anxiety and heightened self-confidence. These findings corroborate previous research that has emphasised these associations across various sports [44,45,46].

Furthermore, the results indicated that a superior sports psychological profile and lower competitive anxiety are also linked with a heightened flow state. This is consistent with prior studies [47,48,49]. Additionally, Hut et al. [50] detailed in their study that mental training, in addition to improving the sports psychological profile and enhancing the connection with the task at hand, reduces athletes’ levels of competitive anxiety, leading to greater well-being and enjoyment in sports practice. These findings align with the present research, underscoring the significance of bolstering the development of psychological resources in young athletes to enhance their well-being and performance in these contexts.

Moreover, older athletes demonstrated a superior psychological profile, competitive anxiety, self-confidence and flow state compared to their younger counterparts. This can be attributed to their greater maturity and accumulated sports experience. Goncalves et al. [35] examined the relationship between maturity and signal-detection skills in a sample of young football players, concluding that more mature players can perceive the game more swiftly and securely, leading to more effective responses. Similarly, López et al. [34] found in their study that players with greater sports experience possessed enhanced cognitive resources to manage stress and potential negative evaluations of their performance.

In addition, players competing at a higher level exhibited a superior psychological profile, competitive anxiety, self-confidence and flow state compared to those competing at a lower level. Castillo-Rodríguez, Hernández-Mendo et al. [51] concluded in their study that athletes at a higher competitive level experience less anxiety and greater self-confidence compared to athletes at a lower competitive level. Athletes who excel are those who effectively use their cognitive and volitional skills [33], so it is likely that this enhanced management of skills allows athletes to reach higher competitive levels.

The psychological aspect is considered as a critical factor in an athlete’s sporting success, alongside technique, tactics and physical ability [52,53]. Based on the results of this study, it is deemed important to focus on psychological skills in the lower categories, as there may be footballers with commendable technical–tactical proficiency and excellent physical condition who could benefit greatly from a fortified psychological profile, enhanced anxiety management, greater self-confidence and an elevated flow state.

Finally, as for the limitations of this study, despite having a substantial sample size, access to an even larger number of athletes could have potentially yielded more precise results for the population. Additionally, the subgroups into which the total sample was divided (14–15 year olds; 16–18 year olds; DH/LN/1st, 2nd/3rd/4th; and playing position) did not have an equal number of athletes. Therefore, for future interventions, it would be beneficial to increase the sample size and strive for a more balanced distribution of athletes across each subgroup to obtain more comprehensive results. Moreover, including under 14 categories in future studies could offer valuable insights into potential differences compared to the analysed categories. Finally, an issue that has not been analysed and that could be important is the starting or substitute status of the athletes and the time each of them plays. This could affect psychological issues such as self-confidence, the athletes’ coping skills or their level of motivation. Therefore, it is suggested to incorporate these analyses in future studies to explore how these psychological variables behave.

## 5. Conclusions

The results obtained reveal that the psychological sports profile is positively related to self-confidence and the flow state. Likewise, competitive anxiety is negatively related to the psychological sports profile, self-confidence and the state of flow. On the other hand, the results show better scores in the analysed variables in favour of the older age category and competitive level, although not by positions on the playing field. This suggests the need to analyse these variables in athletes, with the aim of understanding their profile and being able to intervene with them to improve their psychological resources to face competition.

## Figures and Tables

**Table 1:** Descriptive statistics and Spearman test correlation for sports psychological profile (IPED), competitive anxiety (CSAI-2R), self-confidence (IPED/CSAI-2R) and the flow state (FSS) in young football players.

	M	SD	1	2	3	4	5	6	7	8	9	10	11	12	13	14	15	16	17	18
1. AC (IPED)	4.01	0.62	-																	
2. CAN (IPED)	3.45	0.61	0.45 ^c^	-																
3. CAT (IPED)	3.58	0.58	0.42 ^c^	0.58 ^c^	-															
4. CVI (IPED)	3.69	0.71	0.35 ^c^	0.16 ^b^	0.19 ^b^	-														
5. NM (IPED)	4.24	0.57	0.53 ^c^	0.27 ^c^	0.29 ^c^	0.47 ^c^	-													
6. CAP (IPED)	4.09	0.55	0.65 ^c^	0.49 ^c^	0.47 ^c^	0.45 ^c^	0.63 ^c^	-												
7. CACT (IPED)	3.93	0.57	0.67 ^c^	0.50 ^c^	0.53 ^c^	0.45 ^c^	0.58 ^c^	0.68 ^c^	-											
8. AN-C (CSAI-2R)	2.93	0.66	−0.21 ^c^	−0.45 ^c^	−0.38 ^c^	0.04	0.07	−0.12 ^a^	−0.19 ^b^	-										
9. AN-S (CSAI-2R)	1.80	0.53	−0.21 ^c^	−0.50 ^c^	−0.43 ^c^	−0.06	−0.17 ^b^	−0.30 ^c^	−0.30 ^c^	0.38 ^c^	-									
10. CONF (CSAI-2R)	3.36	0.58	0.69 ^c^	0.45 ^c^	0.42 ^c^	0.37 ^c^	0.49 ^c^	0.62 ^c^	0.65 ^c^	−0.25 ^c^	−0.28 ^c^	-								
11. HD (FSS)	3.86	0.78	0.52 ^c^	0.41 ^c^	0.33 ^c^	0.33 ^c^	0.39 ^c^	0.48 ^c^	0.52 ^c^	−0.19 ^b^	−0.19 ^b^	0.59 ^c^	-							
12. AC (FSS)	3.63	0.79	0.31 ^c^	0.19 ^b^	0.11 ^a^	0.25 ^c^	0.23 ^c^	0.31 ^c^	0.34 ^c^	−0.04	0.01	0.36 ^c^	0.55 ^c^	-						
13. MC (FSS)	4.19	0.72	0.45 ^c^	0.39 ^c^	0.38 ^c^	0.39 ^c^	0.49 ^c^	0.56 ^c^	0.58 ^c^	−0.06	−0.29 ^c^	0.59 ^c^	0.71 ^c^	0.49 ^c^	-					
14. CDC (FSS)	3.85	0.82	0.44 ^c^	0.33 ^c^	0.28 ^c^	0.28 ^c^	0.41 ^c^	0.43 ^c^	0.47 ^c^	−0.11	−0.13 ^a^	0.53 ^c^	0.72 ^c^	0.55 ^c^	0.70 ^c^	-				
15. CT (FSS)	4.13	0.77	0.41 ^c^	0.38 ^c^	0.44 ^c^	0.31 ^c^	0.44 ^c^	0.54 ^c^	0.55 ^c^	−0.11 ^a^	−0.31 ^c^	0.50 ^c^	0.65 ^c^	0.44 ^c^	0.71 ^c^	0.57 ^c^	-			
16. SC (FSS)	3.94	0.79	0.45 ^c^	0.38 ^c^	0.32 ^c^	0.32 ^c^	0.41 ^c^	0.49 ^c^	0.52 ^c^	−0.14 ^a^	−0.20 ^c^	0.56 ^c^	0.74 ^c^	0.58 ^c^	0.75 ^c^	0.75 ^c^	0.71 ^c^	-		
17. PC (FSS)	3.41	0.97	0.35 ^c^	0.36 ^c^	0.24 ^c^	0.23 ^c^	0.21 ^c^	0.30 ^c^	0.33 ^c^	−0.31 ^c^	−0.17 ^b^	0.45 ^c^	0.51 ^c^	0.49 ^c^	0.41 ^c^	0.44 ^c^	0.38 ^c^	0.50 ^c^	-	
18. DT (FSS)	3.11	0.91	0.11 ^a^	−0.04	−0.05	0.10	0.09	0.04	0.03	0.07	0.24 ^c^	0.07	0.17 ^b^	0.33 ^c^	0.08	0.27 ^c^	0.09	0.18 ^b^	0.17 ^b^	-
19. EA (FSS)	4.02	0.92	0.29 ^c^	0.26 ^c^	0.21 ^c^	0.26 ^c^	0.35 ^c^	0.37 ^c^	0.34 ^c^	−0.05	−0.07	0.36 ^c^	0.57 ^c^	0.43 ^c^	0.54 ^c^	0.64 ^c^	0.55 ^c^	0.57 ^c^	0.33 ^c^	0.23 ^c^

^a^ *p* < 0.05; ^b^ *p* < 0.01; and ^c^ *p* < 0.001. M = mean; SD = standard deviation; AC = self-confidence; CAN = negative coping; CAT = attentional control; CVI = visuoimaginative; NM = motivational level; CAP = positive coping; CACT = attitudinal control; AN-C = cognitive anxiety; AN-S = somatic anxiety; CONF = self-confidence; HD = ability level and challenge; AC = action and challenge; MC = clear goals; CDC = direct and clear feedback; CT = concentration on the task; SC = sense of control; PC = loss of self-consciousness; DT = distortion of the sense of time; and EA = autotelic experience.

**Table 2:** Descriptive statistics of the studied variables from the clusters.

	Group 1 (*n* = 140)		Group 2 (*n* = 188)		
	M	DT	M	SD	Z
IPED					
Self-confidence	3.69	0.59	4.25	0.52	−8.31 ***
Negative coping	3.20	0.56	3.64	0.57	−6.36 ***
Attentional control	3.27	0.52	3.63	0.57	−5.97 ***
Visuoimaginative	3.39	0.62	3.90	0.69	−6.53 ***
Motivational level	3.92	0.59	4.48	0.43	−8.37 ***
Positive coping	3.77	0.54	4.33	0.42	−9.12 ***
Attitudinal control	3.60	0.53	4.17	0.46	−9.20 ***
CSAI-2R					
Cognitive anxiety	1.91	0.55	1.71	0.50	−2.06 *
Somatic anxiety	3.02	0.59	2.85	0.70	−3.33 **
Self-confidence	3.02	0.56	3.61	0.44	−9.39 ***
FSS					
Ability and challenge	3.20	0.56	4.36	0.52	−13.39 ***
Action and challenge	3.14	0.60	3.99	0.71	−10.12 ***
Clear goals	3.61	0.63	4.63	0.40	12.96 ***
Direct and clear feedback	3.15	0.65	4.37	0.46	−13.90 ***
Concentration on the task	3.52	0.71	4.58	0.43	−12.43 ***
Sense of control	3.27	0.62	4.45	0.46	−13.92 ***
Loss of self-consciousness	2.87	0.80	3.82	0.88	−8.93 ***
Distortion of time	2.90	0.69	3.26	1.02	−3.73 ***
Autotelic experience	3.35	0.88	4.53	0.56	−11.90 ***

* *p* < 0.05; ** *p* < 0.01; and *** *p* < 0.001.

**Table 3:** Variables in the equation of the binary logistic regression model.

		B	Standard Error	Wald	gl	Sig.	Exp(B)	95% C.I. to Exp(B)	
								Lower	Upper
Step 1	Self-confidence. CSAI-2R	2.240	0.277	65.523	1	0.000	9.393	5.461	16.156
	Constant	−7.214	0.940	58.949	1	0.000	0.001		
Step 2	Motivational level. IPED	1.435	0.284	25.471	1	0.000	4.202	2.406	7.337
	Self-confidence. CSAI-2R	1.679	0.292	33.067	1	0.000	5.360	3.024	9.499
	Constant	−11.409	1.365	69.901	1	0.000	0.000		
Step 3	Motivational level. IPED	1.097	0.307	12.754	1	0.000	2.995	1.640	5.468
	Attitudinal control. IPED	1.071	0.370	8.399	1	0.004	2.918	1.414	6.021
	Self-confidence. CSAI-2R	1.260	0.322	15.313	1	0.000	3.527	1.876	6.630
	Constant	−12.762	1.511	71.316	1	0.000	0.000		
Step 4	Negative coping. IPED	0.570	0.283	4.047	1	0.044	1.767	1.015	3.078
	Motivational level. IPED	1.144	0.310	13.653	1	0.000	3.140	1.711	5.762
	Attitudinal control. IPED	0.845	0.386	4.789	1	0.029	2.327	1.092	4.960
	Self-confidence. CSAI-2R	1.136	0.327	12.072	1	0.001	3.115	1.641	5.912
	Constant	−13.597	1.599	72.315	1	0.000	0.000		

**Table 4:** Descriptives of sports psychological profile (IPED), competitive anxiety (CSAI-2R), self-confidence (IPED/CSAI-2R) and flow state (FSS) based on age category and competitive level.

	Age Category					Competitive Level				
	14–15 Year Olds (*n* = 172)		16–18 Year Olds (*n* = 156)			DH/LN/1ª (*n* = 138)		2ª/3ª/4ª (*n* = 190)		
	M	SD	M	SD	Z	M	SD	M	SD	Z
IPED										
Self-confidence	3.92	0.62	4.11	0.59	−2.86 **	4.18	0.51	3.89	0.65	−4.10 ***
Negative coping	3.40	0.63	3.50	0.58	−1.64	3.57	0.56	3.36	0.62	−3.17 **
Attentional control	3.44	0.59	3.52	0.56	−1.28	3.51	0.58	3.45	0.57	−1.25
Visuoimaginative	3.61	0.73	3.77	0.68	−2.00 *	3.80	0.66	3.60	0.74	−2.36 *
Motivational level	4.27	0.55	4.20	0.59	−1.00	4.33	0.48	4.17	0.63	−1.86
Positive coping	4.07	0.55	4.12	0.56	−0.94	4.21	0.49	4.01	0.58	−3.02 **
Attitudinal control	3.86	0.57	4.00	0.56	−2.23 *	4.06	0.48	3.83	0.61	−3.54 ***
CSAI-2R										
Cognitive anxiety	3.00	0.66	2.85	0.65	−2.09 *	2.87	0.64	2.97	0.67	−1.52
Somatic anxiety	1.81	0.56	1.78	0.50	−0.14	1.68	0.48	1.88	0.55	−3.27 **
Self-confidence	3.27	0.58	3.47	0.56	−3.56 ***	3.53	0.49	3.24	0.60	−4.65 ***
FSS										
Ability and challenge	3.72	0.79	4.01	0.74	−3.42 **	4.11	0.71	3.68	0.78	−4.91 ***
Action and challenge	3.49	0.78	3.78	0.77	−3.68 ***	3.67	0.86	3.60	0.73	−0.91
Clear goals	4.13	0.73	4.26	0.71	−1.80	4.37	0.65	4.06	0.74	−3.87 ***
Direct and clear feedback	3.75	0.82	3.96	0.80	−2.43 *	4.04	0.75	3.70	0.84	−3.67 ***
Concentration on the task	4.07	0.80	4.20	0.73	−1.39	4.26	0.73	4.03	0.78	−2.81 **
Sense of control	3.82	0.82	4.08	0.73	−2.83 **	4.10	0.79	3.84	0.77	−3.36 **
Loss of self-consciousness	3.34	0.95	3.49	0.98	−1.49	3.48	0.92	3.36	1.00	−1.08
Distortion of time	3.07	0.88	3.14	0.94	−0.48	3.04	0.93	3.15	0.90	−88
Autotelic experience	3.98	0.95	4.08	0.90	−1.07	4.03	0.99	4.02	0.87	−87

* *p* < 0.05; ** *p* < 0.01; and *** *p* < 0.001. DH/LN/1ª = Division of Honor/National League/1st Andalusian; 2ª/3ª/4ª = 2nd/3rd/4th Andalusian.

**Table 5:** Descriptives of sports psychological profile (IPED), competitive anxiety (CSAI-2R), self-confidence (IPED/CSAI-2R) and flow state (FSS) based on the playing position on the field.

	Playing Position on the Field								
	Goalkeeper (*n* = 31)		Defender (*n* = 111)		Midfielder (*n* = 89)		Forward (*n* = 97)		
	M	DT	M	DT	M	DT	M	DT	χ^2^
IPED									
Self-confidence	4.15	0.48	4.06	0.66	3.99	0.62	3.94	0.60	4.02
Negative coping	3.53	0.67	3.49	0.56	3.46	0.68	3.37	0.56	2.84
Attentional control	3.48	0.57	3.52	0.62	3.54	0.56	3.36	0.54	7.52
Visuoimaginative	3.73	0.88	3.71	0.71	3.61	0.65	3.71	0.70	1.82
Motivational level	4.41	0.57	4.22	0.57	4.20	0.60	4.24	0.55	4.00
Positive coping	4.12	0.55	4.12	0.57	4.13	0.55	4.02	0.54	2.90
Attitudinal control	4.02	0.58	3.96	0.58	3.93	0.55	3.87	0.57	2.63
CSAI-2R									
Cognitive anxiety	2.88	0.66	2.93	0.63	2.80	0.69	3.04	0.65	2.22
Somatic anxiety	1.76	0.49	1.85	0.56	1.73	0.54	1.81	0.49	6.45
Self-confidence	3.48	0.50	3.40	0.59	3.35	0.60	3.29	0.56	4.07
FSS									
Ability and challenge	3.86	0.93	3.91	0.78	3.87	0.73	3.80	0.79	1.03
Action and challenge	3.64	0.89	3.64	0.81	3.58	0.78	3.66	0.74	0.42
Clear goals	4.23	0.81	4.19	0.75	4.22	0.65	4.16	0.72	0.48
Direct and clear feedback	4.12	0.81	3.84	0.80	3.78	0.85	3.83	0.81	4.19
Concentration on the task	4.04	0.87	4.17	0.77	4.17	0.76	4.08	0.75	1.43
Sense of control	4.01	0.85	3.93	0.79	3.90	0.78	3.98	0.79	0.86
Loss of self-consciousness	3.34	1.08	3.52	0.92	3.28	1.02	3.44	0.94	2.79
Distortion of time	3.09	0.87	3.08	0.88	3.02	0.97	3.22	0.91	1.67
Autotelic experience	4.23	0.91	4.09	0.88	3.94	0.95	3.98	0.95	3.57

**χ^2^** = Chi-square.

## Data Availability

Data are available upon request to the authors.
